# The adapter protein Myd88 plays an important role in limiting mycobacterial growth in a zebrafish model for tuberculosis

**DOI:** 10.1007/s00428-021-03043-3

**Published:** 2021-02-09

**Authors:** Rohola Hosseini, Gerda E. M. Lamers, Erik Bos, Pancras C. W. Hogendoorn, Abraham J. Koster, Annemarie H. Meijer, Herman P. Spaink, Marcel J. M. Schaaf

**Affiliations:** 1 Institute of Biology Leiden, Leiden University, Leiden, Netherlands; 2 Department of Cell and Chemical Biology, Leiden University Medical Center, Leiden, Netherlands; 3 Department of Pathology, Leiden University Medical Center, Albinusdreef 2, 2333 Leiden, ZA Netherlands

**Keywords:** Zebrafish, Tail fin, Infection, Mycobacterium, Tuberculosis, Macrophage, Leukocyte dynamics, Efferocytosis, Cell death

## Abstract

Tuberculosis (TB) is the most prevalent bacterial infectious disease in the world, caused by the pathogen *Mycobacterium tuberculosis* (*Mtb*). In this study, we have used *Mycobacterium marinum* (*Mm*) infection in zebrafish larvae as an animal model for this disease to study the role of the myeloid differentiation factor 88 (Myd88), the key adapter protein of Toll-like receptors. Previously, Myd88 has been shown to enhance innate immune responses against bacterial infections, and in the present study, we have investigated the effect of Myd88 deficiency on the granuloma morphology and the intracellular distribution of bacteria during *Mm* infection. Our results show that granulomas formed in the tail fin from *myd88* mutant larvae have a more compact structure and contain a reduced number of leukocytes compared to the granulomas observed in wild-type larvae. These morphological differences were associated with an increased bacterial burden in the *myd88* mutant. Electron microscopy analysis showed that the majority of *Mm* in the *myd88* mutant are located extracellularly, whereas in the wild type, most bacteria were intracellular. In the *myd88* mutant, intracellular bacteria were mainly present in compartments that were not electron-dense, suggesting that these compartments had not undergone fusion with a lysosome. In contrast, approximately half of the intracellular bacteria in wild-type larvae were found in electron-dense compartments. These observations in a zebrafish model for tuberculosis suggest a role for Myd88-dependent signalling in two important phenomena that limit mycobacterial growth in the infected tissue. It reduces the number of leukocytes at the site of infection and the acidification of bacteria-containing compartments inside these cells.

## Introduction

Pulmonary tuberculosis (TB) is an often-lethal bacterial infection caused by *Mycobacterium tuberculosis* (*Mtb*), which is estimated to have infected one-third of the global population. Currently, over a million people die as a consequence of this infection annually [1]. Increasing occurrence of multi-drug-resistant *Mtb* strains is widespread, and in order to develop novel therapeutic strategies, a better understanding of tuberculosis is required [2, 3].

During the pathogenesis of TB, *Mtb* displays a complex interaction with the immune system of the host. *Mtb* is phagocytized by macrophages, in which it prevents acidification and degradation of the phagosomal content [4, 5]. In addition, there is evidence that *Mtb* is able to escape from the phagosomes into the cytoplasm of the macrophage [6, 7]. In these infected cells, the *Mtb* bacteria create a niche, in which they can survive for long periods of time and replicate [8, 9]. Pro-inflammatory signals from infected macrophages initiate the recruitment of other innate and adaptive immune cells to the primary infection site, leading to the formation of highly organized granulomatous lesions. In these granulomas, *Mtb* can persist for many years, forming a latent infection by minimizing its metabolic and replicative activity. However, *Mtb* can also be reactivated resulting in an active TB infection [10].

Despite the fact that *Mtb* exploits macrophages to persist inside its host, this cell type is indispensable for the host in order to keep *Mtb* infection under control. Macrophages recognize invading pathogens at the first stage of infection and initiate an immune response. Recognition of pathogen-associated molecular patterns (PAMPs) and endogenous danger-associated molecular patterns (DAMPs) occurs through pattern recognition receptors (PRRs), of which the Toll-like receptors (TLR) are one of the major classes [11, 12]. Myeloid differentiation factor 88 (MYD88) is a key adaptor protein in the TLR signalling pathway since it is used by all TLRs (except for TLR3) to initiate an inflammatory response [13]. The C-terminal TIR domain of MYD88 enables interaction with TLRs or the interleukin-1 receptor (IL1R), and the N-terminal death domain enables the formation of a “Myddosome” signalling complex, consisting of IL-1 receptor associated kinases (IRAKs). The Myddosome plays a central role in inflammation and host defence by activating the mitogen-activated protein kinase (MAPK) signalling pathway and the nuclear factor-ĸB (NF-ĸB) transcription factor complex [14–17]. MyD88-deficient mice show increased susceptibility to various pathogens, among them *Mtb* [18].

Zebrafish are naturally susceptible to mycobacterial infection, caused by *Mycobacterium marinum* (*Mm*), which is genetically closely related to *Mtb*. *Mm* induces a similar pathology to its human equivalent, including the formation of tuberculous granulomas [19, 20]. The larval stage of the zebrafish enables detailed in vivo imaging and has been used extensively to study host-pathogen interactions during *Mm* infection [19, 21–23]. In zebrafish larvae, infected macrophages and neutrophils aggregate and form initial granulomas, which makes this model highly suitable to study the role of innate immune cells during the progression of mycobacterial infection [24]. The observed granulomas at larval stage appear to be highly dynamic in nature, characterized by the active recruitment of macrophages during early *Mm* infection and the reverse migration of infected macrophages from infected sites [25].

In previous work, we have shown that the Myd88-signalling pathway has a protective role during *Mm* infection in zebrafish larvae [26–29]. Larvae from a *myd88* mutant line (*myd88*^*-/-*^) or *myd88* knockdown larvae showed decreased induction of pro-inflammatory cytokines [28, 30], lower production of reactive nitrogen species by neutrophils [26] and attenuated initiation of autophagic defence [27], resulting in increased rates of infection.

In the present study, we have used the *myd88*^*-/-*^ line to study the effect of Myd88 deficiency on granuloma morphology and subcellular localization of *Mm* infection. To this end, we used our previously described tail fin injection model [24, 31], in which the formation of a single granuloma can be monitored over time and imaged by a combination of confocal and electron microscopy. Our results show that *Mm* infection in *myd88* mutant larvae results in an increased bacterial burden associated with strongly reduced recruitment of leukocytes to granulomas. The majority of *Mm* was found to be located extracellularly in *myd88* mutants, and bacteria that were found inside cells were mostly observed as aggregates in compartments that were not acidified. These data indicate a specific role for Myd88-dependent signalling in the protection against *Mm* infection.

## Materials and methods

### Zebrafish strains and maintenance

Zebrafish were handled in compliance with the local animal welfare regulations and maintained according to standard protocols (www.zfin.org). Wild-type and the *myd88*^hu3568^ *z*ebrafish strains were used for this study, and culturing the *myd88*^hu3568^ strain was approved by the local animal welfare committee (DEC) of the University of Leiden (protocol 12232). All fish were raised and grown at 28.5 °C on a 14-h light:10-h dark cycle. Embryos were obtained from natural spawning at the beginning of the light period and kept in egg water (60 μg/ml Instant Ocean sea salts).

### Zebrafish tail fin infection

**Fig. 1.**
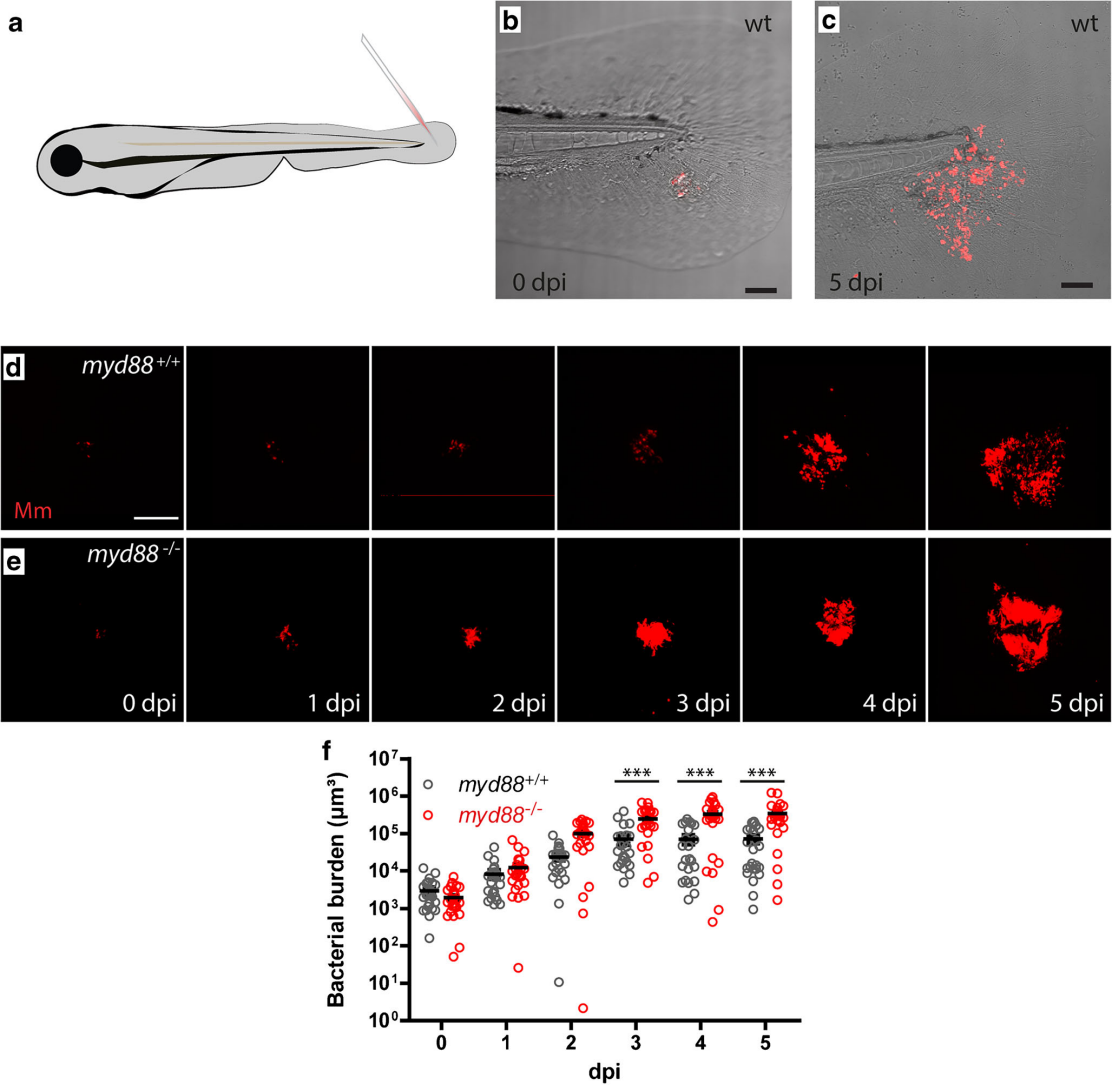
Granuloma development and morphology in the tail fin of zebrafish larvae. **a** *Mm* injection in the tail fin generates single granuloma. **b**,**c** Infected tail fin of the same larva with fluorescently labelled *Mm* (red) at 4 h post infection (**b**) and at 4 days post infection (**c**) showing the localized development of the early granuloma structures. **d,e** Representative images of larvae showing the increase of *Mm* infection (red) and development of granuloma structures in the wild type (**d**) and *myd88*^-/-^ larvae (**e**). **f** Bacterial burden in the *myd88*^+/+^ (black) and *myd88*^-/-^ (red) larvae. The data (mean ± SEM) were analysed using analysis of variance (ANOVA); Bonferroni’s multi comparison post-test was performed on *myd88* wild type and mutant larvae at each time point (***p < 0.0001, *n* > 20 larvae per time point). The scale bars represent 100 μm

The *M. marinum* M strain fluorescently labelled with E2-crimson was used and prepared at ~ 50 colony-forming units per 1 nl as previously described [32]. Borosilicate glass micro-capillaries (Harvard Apparatus, USA) were used with a micropipette puller device (Sutter Instruments Inc., USA) for preparing microinjection needles. Zebrafish larvae were injected in the tail fin at 3 dpf using the Eppendorf microinjection system with a fine (~ 5 to 10 micron) needle tip broken off with tweezers and mounted at a 30-degree angle. Larvae were anesthetized in egg water with 200 μg/mL 3-aminobenzoic acid (tricaine; Sigma-Aldrich, USA) and injected between the 2 epidermal layers at the ventral part of the tail fin (Fig. 1), as previously described [31]. Larvae were fixed at desired time points after infection with 4% paraformaldehyde in PBS-T (phosphate-buffered saline; NaCl 150 mM, K_2_HPO_4_ 15 mM, KH_2_PO_4_ 5 mM) with 0.05% Tween 20 (Merck Millipore, Germany) with gentle agitation for 18 h at 4 °C. The larvae were washed the next day with PBS-T and stored at 4 °C for further staining or until imaging.

### Lcp1 immunohistochemistry

For Lcp1 immunostaining, larvae were anesthetized with 200 μg/ml tricaine and then immediately fixated in 4% paraformaldehyde in PBS (phosphate-buffered saline, pH 7.2) for 16 h at 4 °C. After fixation, the larvae were rinsed in PBS-DTx (phosphate-buffered saline with 0.5% DMSO and 0.3% Triton X-100) and treated with proteinase K (10 μg/ml in PBS-DTx; Roche) for 10 min at 37 °C. The larvae were blocked in 5% normal sheep serum (Sigma-Aldrich) in PBS-DTx for 2 h at room temperature, incubated with Lcp1/L-Plastin antibody (a gift from Dr. Anna Huttenlocher, University of Wisconsin, USA) in 1:1000 dilution at 4 °C overnight and subsequently incubated with Alexa-488 conjugated secondary antibody (1:200; Invitrogen) for 2 h at room temperature. The larvae were washed with PBS-DTx and stored at 4 °C until imaging.

### TUNEL assay

The TUNEL experiments were performed after fixation (anesthetized with 200 μg/ml tricaine and afterwards immediately fixated in 4% paraformaldehyde in PBS for 16 h at 4 °C) of larvae using Millipore ApopTag Peroxidase In Situ Apoptosis detection kit and Roche Anti-Digoxigenin-POD Fab fragments using a protocol adapted for use in zebrafish larvae [33]. TSA Fluorescence kits (Perkin Elmer, USA) Fluorescein was used for fluorescence detection.

### Confocal laser scanning microscopy

**Fig. 2:**
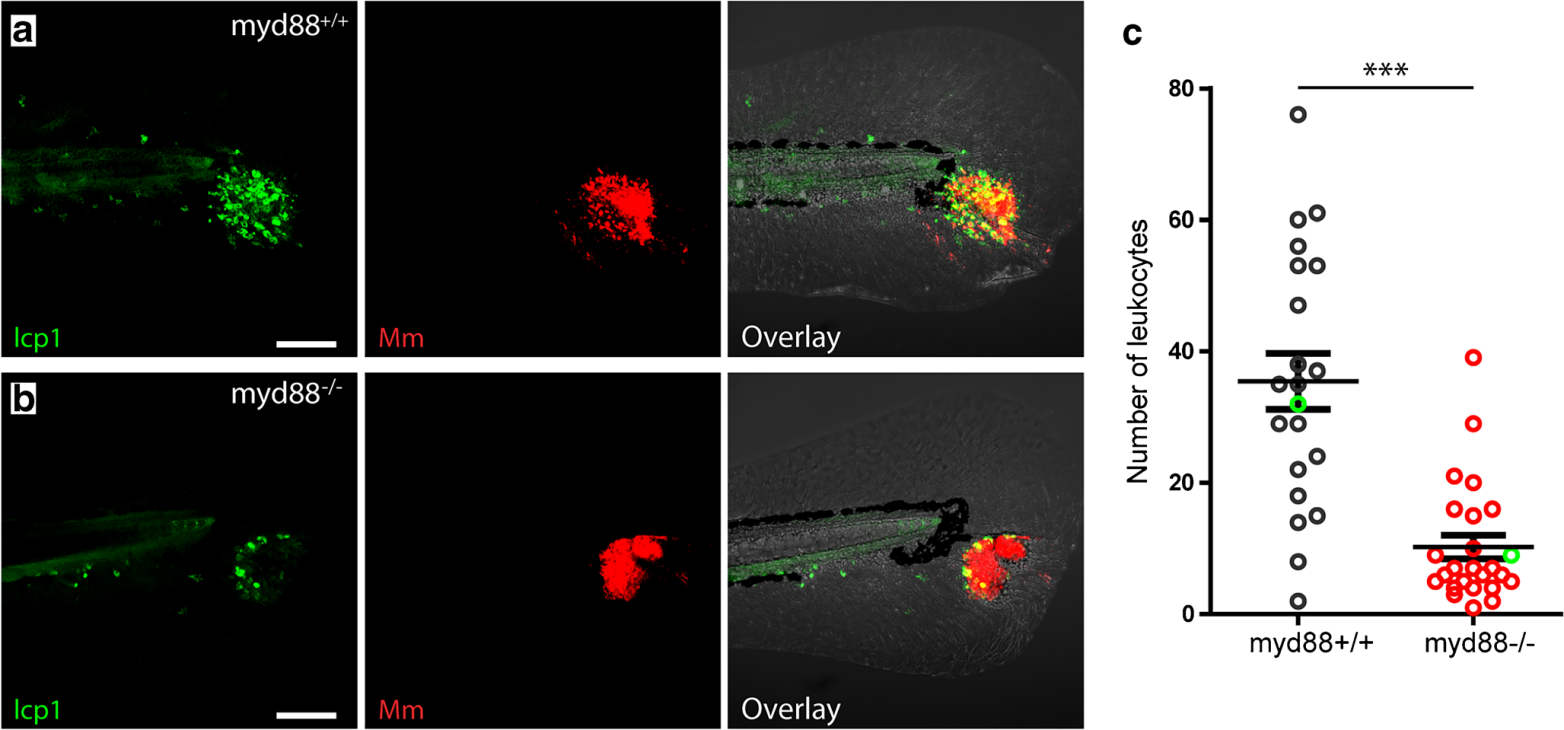
Number of leukocytes at the site of infection in *myd88*^+/+^ and *myd88*^-/-^ larvae. **a,b** Representative images of a larva showing lcp1-positive cells (green) and *Mm* (red) in *myd88*^+/+^ (**a**) and *myd88*^-/-^ (**b**) larvae at 4 dpi. **c** Quantification of lcp1-positive cell shows less leukocytes to be present at the site of infection in *myd88*^-/-^ larvae. The data (mean ± SEM) were analysed using a two-tailed student *t* test (***p < 0.001, *n* > 20 larvae per condition). Scale bar represents 100 μm

**Fig. 3:**
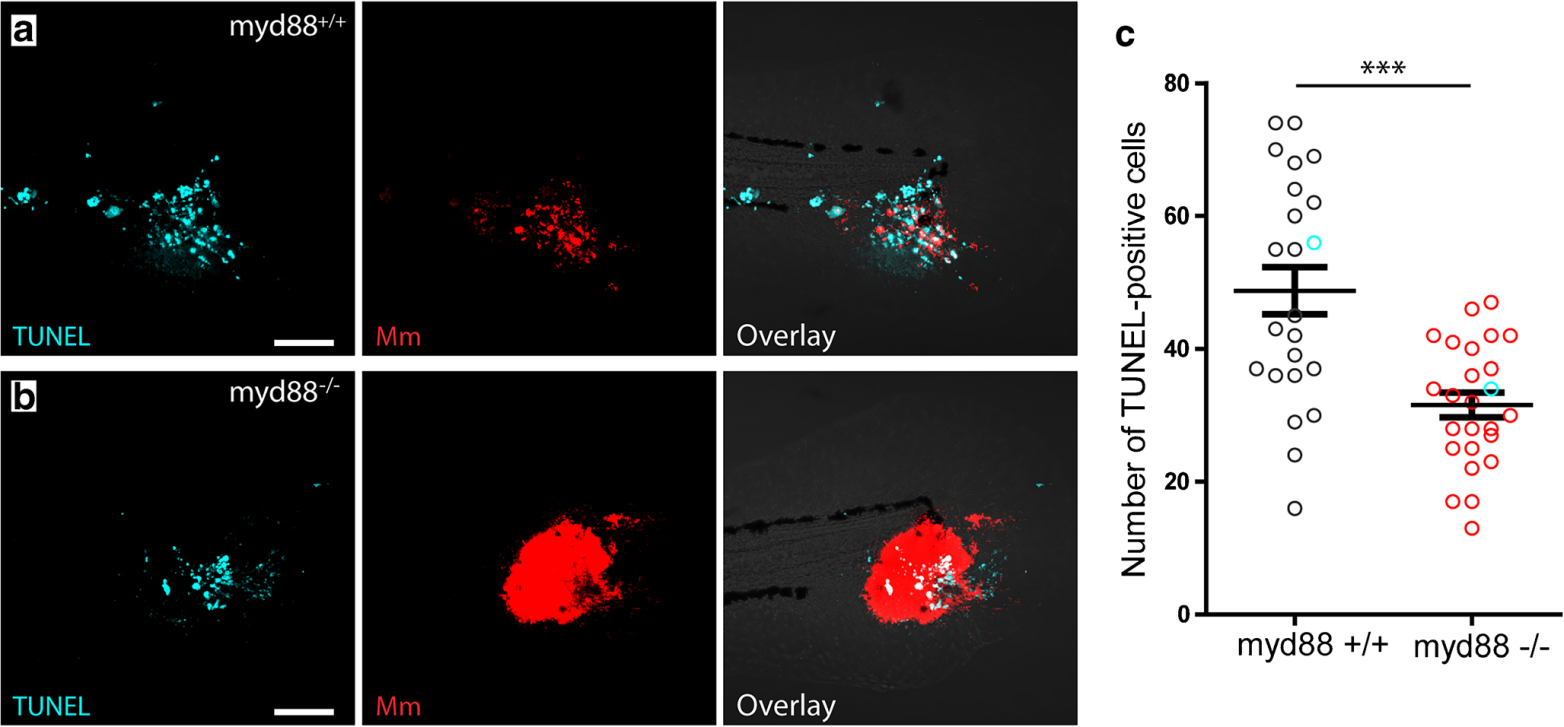
TUNEL-positive cells at the site of infection in *myd88*^+/+^ and *myd88*^-/-^ larvae. **a,b** Representative images of a larva showing TUNEL-positive cells (green) and *Mm* (red) in *myd88*^+/+^ (**a**) and *myd88*^-/-^ (**b**) larvae at 4 dpi. **c** Quantification of TUNEL-positive cell shows fewer dead cells to be present at the site of infection in *myd88*^-/-^ larvae. The data (mean ± SEM) were analysed using a two-tailed student *t* test (***p < 0.001, *n* > 20 larvae per condition). Scale bar represents 100 μm

Larvae were mounted in 1% low melting agarose (Sigma-Aldrich, USA) and imaged with a Leica TCS SPE (Wetzlar, Germany) confocal laser scanning microscope (CLSM) using 488 and the 633 laser lines and a 20X (NA 0.7) objective (for the images used in Fig. 1b–e) or a Nikon A1 CLSM (Tokyo, Japan) using 488 and the 641 laser lines and a 20X (NA 0.75) objective (for the images used in Figs. 1f, 2 and 3). Images were analysed using Fiji [34] or NIS-elements 4 (Nikon) software. The images of larvae used in Fig. 1b–e were visually inspected using maximum z-projections, and cropped regions of infection were generated using Fiji software. The bacterial burden was measured as the total volume (in μm^3^) of the fluorescent signal of *M. marinum* in the 3D images (12 bit) in Nis-elements 4, i.e. the total volume of the voxels with a signal above a certain fluorescence intensity threshold (using the same threshold for all larvae). The numbers of TUNEL- and Lcp1-positive cells were counted manually for each image.

### Transmission electron microscopy

Before being used for electron microscopy, the zebrafish larvae were anesthetized with 200 μg/ml tricaine and afterwards immediately fixated in 2% glutaraldehyde and 2% paraformaldehyde in sodium cacodylate buffer (pH 7.2) for 3 h at room temperature followed by fixation for 16 h at 4 °C. Postfixation was performed in 1% osmium tetroxide in sodium cacodylate buffer for 1 h at room temperature. After dehydration through a graded series of ethanol, all specimens were kept in epoxy resin (Agar Scientific, UK) for 16 h before embedding. Ultra-thin sections were collected on Formvar coated 200 mesh or one-hole copper grids (Agar Scientific, UK) stained with 2% uranyl acetate in 50% ethanol and lead citrate for 10 min each. Electron microscopy images were obtained with a JEM-1010 transmission electron microscope (JEOL, Japan) equipped with an Olympus Megaview camera (Tokyo, Japan). A 200 kV Tecnai 20 FEG transmission electron microscope (FEI Company, the Netherlands) equipped with a 4 k × 4 K CCD camera (Gatan, USA) was used to image large specimen tissues with the automatic montaging of individual images into large overviews with high resolution [35].

For TEM imaging and a quantitative analysis of the images, three larvae for each group (wild type and mutant) were used, and a section in the middle of the initial granulomas was imaged. Quantification was done by manually counting and classifying individual bacteria. The total number of bacteria counted for this analysis was 2178 and 1655, for wild type and mutant, respectively.

### Statistical analysis

All data (mean ± SEM) were analysed using Prism version 5.0 (GraphPad Software) using one-way analysis of variance (ANOVA) with Bonferroni’s multi comparison post-test for multiple groups. Two-tailed Student *t* tests were used for comparing 2 conditions.

## Results

### Myd88 deficiency affects granuloma morphology and increases bacterial burden

We have previously shown that TLR/IL1R-Myd88 signalling is required for defence of zebrafish larvae systemically infected with *M. marinum* (*Mm*). In the present study, we used a previously established localized infection in the tail fin [31], to investigate the initial granuloma formation and subcellular localization of *Mm* at the site of infection. Homozygous *myd88* mutant (*myd88*^-/-^) and wild-type larvae (*myd88*^+/+^) were infected with ~ 50 colony-forming units (cfu) of fluorescently labelled *Mm* in the tail fin at 3 days post fertilization (dpf). The infection in these larvae develops into a granuloma-like structure within 3 to 5 days post infection (dpi).

To provide a detailed description of the infection process and the development of the granuloma structure, confocal laser scanning microscopy (CLSM) was performed on the tail fin of infected *myd88*^-/-^ and wild-type (*myd88*^+/+^) larvae. The bacterial burden in representative *myd88*^+/+^ and *myd88*^-/-^ larvae is shown in Fig. 1. The infection in each of these larvae was imaged at 4 h post infection (hpi) and at 1, 2, 3, 4 and 5 dpi. From 2dpi onward we observed compacted bacterial aggregates in *myd88*^-/-^ larvae (Fig. 1e), while in the *myd88*^+/+^ larvae, these aggregates were smaller and more widely distributed over the infected tissue (Fig. 1d).

In a separate experiment, the bacterial burden was quantified in the *myd88*^+/+^ and *myd88*^-/-^ larvae, based on fixed samples at 0 to 5 dpi (Fig. 1F). The bacterial burden was measured as the total volume of the fluorescent signal of *Mm*. The burden increased significantly between 0 and 3 dpi in the *myd88*^+/+^ and the *myd88*^-/-^ larvae but increased at a lower rate in the *myd88*^+/+^ larvae, compared to the rate in the *myd88*^-/-^ larvae, resulting in larger burdens at later time points in the mutant. At 4dpi, the difference in infection level between *myd88*^+/+^ and *myd88*^-/-^ larvae was maximal with a bacterial volume of 69·10^3^ (± 1.6·10^3^) μm^3^ and 328·10^3^ (± 52·10^3^) μm^3^, respectively. In line with previous results obtained using a systemic infection model [28], localized *Mm* infection in the tail fin of *myd88*^-/-^ larvae results in an increased bacterial burden.

### Reduced number of leukocytes at the infection site in *myd88*^*-/-*^ larvae

In order to investigate the presence of leukocytes at the site of infection in *myd88*^-/-^ zebrafish, we performed Lcp1/L-plastin immunostaining at 4 dpi for visualization of all leukocytes (Fig. 2). The larvae were imaged using CLSM, and representative images are shown for *myd88*^+/+^ and *myd88*^-/-^ larvae (Fig. 2 a and b, respectively). At 4 dpi, the infection in the tail fin has resulted in the formation of an initial stage granuloma, which in *myd88*^+/+^ larvae was observed as a large local accumulation of L-plastin-positive cells (35.4 ± 4.2) at the site of the infection in the tail fin (Fig. 2a and c). In the *myd88*^-/-^ larvae, the number of L-plastin-positive cells at the site of infection was significantly lower (10.2 ± 1.8).

The lower number of leukocytes observed at the site of infection in *myd88*^-/-^ larvae could either be due to a lower number of leukocytes recruited to the infected area or to a higher rate of cell death of these cells. To address this issue, we performed a fluorescent terminal deoxynucleotidyl transferase dUTP nick end-labelling (TUNEL) assay, which visualizes double-stranded DNA breaks, thereby labelling apoptotic as well as necrotic cells (Fig. 3). At 4 dpi, *myd88*^+/+^ larvae show a high number of TUNEL positive cells (48.7 ± 3.6) throughout the site of infection (Fig. 3a). The *myd88*^-/-^ larvae show a lower number of TUNEL positive cells (31.6 ± 1.8; Fig. 3b and c). Although alternative explanations such as infection-induced Myd88-dependent changes in haematopoiesis, or leukocyte cell death that is occurring systemically at earlier time points, the observations suggest that the lower number of leukocytes at the infection sites in *myd88*^-/-^ larvae is a consequence of reduced recruitment rather than increased cell death.

### Transmission electron microscopy reveals reduced acidification of *Mm* containing compartments in *myd88*^-/-^ larvae

**Fig. 4:**
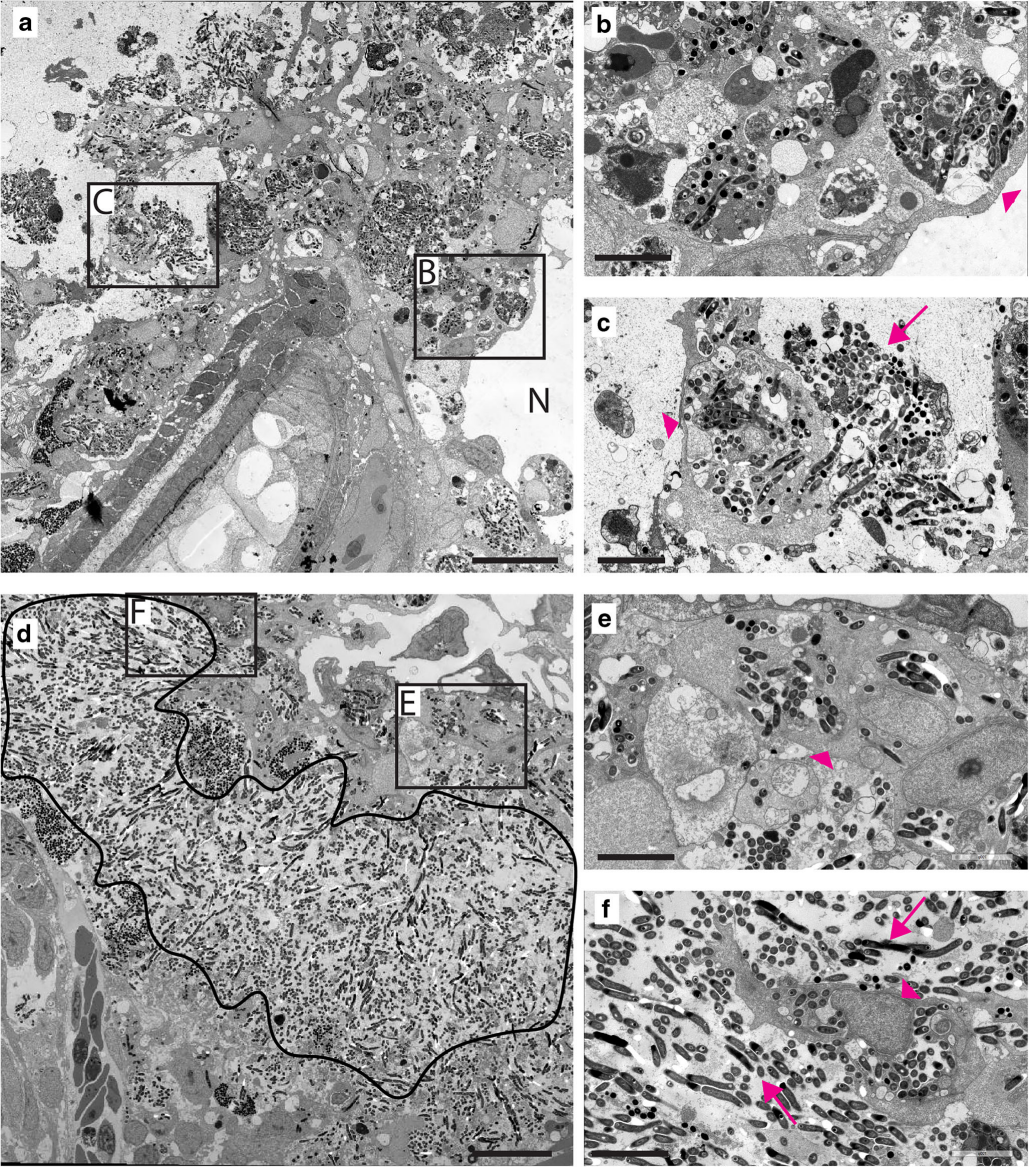
Granuloma structures in *myd88*^*-/-*^ larvae consist mainly of extracellular *Mm*. **a** TEM image of a granuloma in representative *myd88*^+/+^ larvae, showing the necrotic centre (N) and aggregates of *Mm* in the immune cells. **b,c** Higher magnification of regions indicated in (**a)**, showing infected cells (arrowheads) and extracellular *Mm* (arrows). **d** TEM image of a granuloma in representative *myd88*^-/-^ larvae showing the area with extracellular *Mm* (black line). **e,f** Higher magnification of region indicated in (**d**), showing extracellular bacteria and infected cells (arrowheads). *n* = 3 per group, the scale bars in (**a**)–(**d**) (20 μm) and in (**b**)–(**c**) and (**e**)–(**f**) (10 μm)

In order to analyse effects of Myd88 deficiency at an ultrastructural level, transmission electron microscopy (TEM) was performed on the tail fin granulomas of *Mm* infected *myd88*^+/+^ and *myd88*^-/-^ larvae (Fig. 4). At 5 dpi, the *myd88*^+/+^ larvae show a necrotic centre in the infected area, visible as a hole in the tail fin, surrounded by bacteria-containing cells (Fig. 4a and b). In the *myd88*^-/-^ larvae, the majority of bacteria were found to reside extracellularly (Fig. 4c and d), and the area containing extracellular bacteria is surrounded by infected cells (Fig. 4d). Such areas with extracellular bacteria are absent in wild-type larvae.

**Fig. 5.**
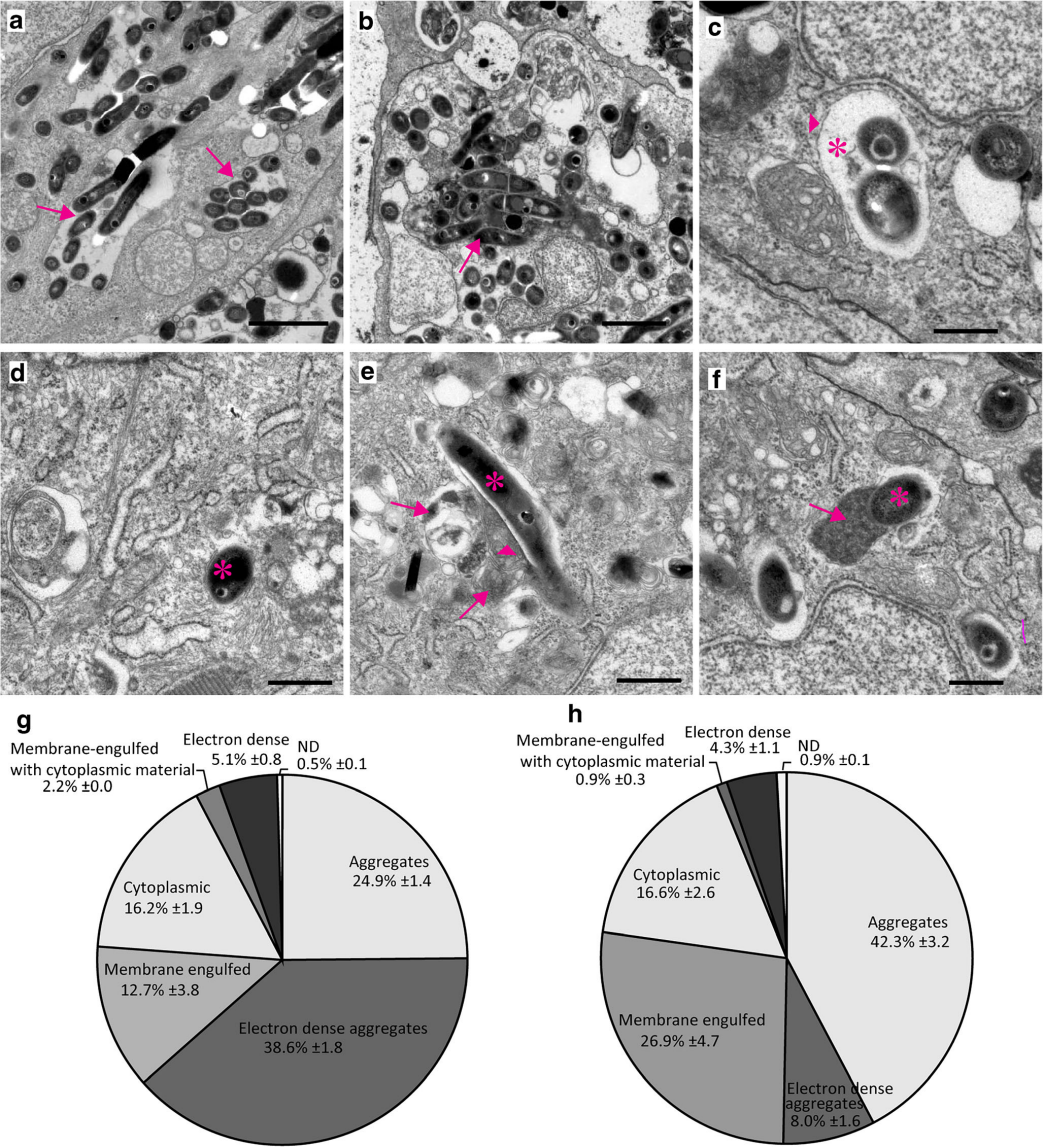
Quantification of intracellular *Mm* shows altered distribution of bacteria in different compartments in *myd88*^*-/-*^ larvae. **a–f** Representative TEM images of *Mm* in different compartments. **a** Aggregates as a compact cluster of bacteria (> 5) without any electron-dense areas (arrows). **b** Electron-dense aggregates as a compact cluster of bacteria in a compartment having a uniform high electron density between the bacteria (arrowhead) and/or electron-dense regions (arrow). **c** Membrane-engulfed compartments containing bacteria surrounded by a single membrane (arrowhead) with an electron-transparent zone (asterisk), without any cytoplasmic material. **d** Cytoplasmic bacteria not enclosed by a membrane, indicated by a white asterisk. **e** Membrane-engulfed compartments with cytoplasmic material containing bacteria (asterisk), and partially degraded content (arrowhead) and other fused vacuoles (arrows). **f** Electron-dense compartments containing bacteria (asterisk) with uniform electron-dense content (arrow). **g,h** The fractions (± SEM) of intracellular *Mm* found in different compartments or free in the cytoplasm is presented in a pie chart for *myd88*^+/+^ (total is 2178 bacteria) (**g**) and *myd88*^-/-^ (total is 1655 bacteria) (**h**). N is 3 larvae with initial stage granulomas per group at 4dpi, with the section at middle of granuloma were imaged and analysed for each larva. The scale bars in (**a**)–(**b**) (2 μm) and in (**c**)–(**f**) (500 nm)

To determine the nature and frequency of different cell-bacterium interactions in *myd88*^+/+^ and *myd88*^-/-^ larvae, we used a previously described procedure [31], in which we quantified the occurrence of intracellular *Mm* in the cytoplasm without any membrane structures surrounding it, or in different types of intracellular compartments, as individual bacteria or as aggregates. The results of this quantification and representative images of each type of interaction are shown in Fig. 5. A large fraction of intracellular bacteria occurred in compartments containing more than 5 bacteria (hereafter referred to as aggregates), both in *myd88*^+/+^ (~ 64%) and in *myd88*^-/-^ larvae (~ 50%) (Fig. 5a and b). However, electron-dense compartments containing these aggregates (hereafter referred to as electron-dense aggregates) were much less abundant in mutant larvae (~ 8%) compared to wild type (~ 39%, Fig. 5b). These compartments are characterized by a uniform electron-dense content and probably reflect lysosomes or phagosomes that have been fused with a lysosome. Individual bacteria were present in membrane-engulfed compartments, characterized by a single membrane without any cytoplasmic material, probably reflecting phagosomal structures (Fig. 5c). In mutant larvae, those membrane-engulfed compartments were found more often (~ 27%) than in the wild types (~ 13%). Bacteria in the cytoplasm, not enclosed by a membrane, were observed in the type and mutant larvae at a similar frequency, (~ 16% and ~ 17% respectively; Fig. 5d). In addition, bacteria were occasionally found in membrane-engulfed compartments containing cytoplasmic material, in the *myd88*^+/+^ (~ 2%) and *myd88*^-/-^ (~ 1%) larvae. These compartments are characterized by partially degraded cytoplasmic content and organelles, probably as a result of engulfment by or fusion with autophagosomes. This morphology was previously shown to be associated with the localization of the Lc3 protein, using correlative light and electron microscopy, which also suggests an autophagic origin [31] (Fig. 5e). Finally, sometimes bacteria were located inside electron-dense, membrane-engulfed compartments with a regular electron-dense content in the wild-type and *myd88*^-/-^ larvae (~ 5% and ~ 4% respectively; Fig. 5f). These electron-dense compartments probably reflect lysosomes with acidic content. In conclusion, the most notable difference between *myd88*^*-/-*^ and wild-type larvae was the reduced presence of electron-dense aggregates in the mutant.

## Discussion

In this study, we provide a new insight in the early stages of granuloma development and ultrastructural morphology during *Mm* infection in zebrafish larvae using both light and electron microscopy. We show that upon injection of bacteria in the tail fin, a localized infection develops in both *myd88*^+/+^ and *myd88*^-/-^ larvae. In the *myd88*^-/-^ larvae, the infection developed more rapidly resulting in an increased infection rate at 4 dpi. This is consistent with our earlier observations of an increased bacterial burden in *myd88*^-/-^ larvae using a blood island infection model [28]. In addition, using the tail fin infection model, we observed a clearly different phenotype of infection in the mutant compared to the wild-type larvae (Fig. 1d and e). The results of the present study suggest that the increased bacterial burden is a result of two compromised host-protective processes: the recruitment of leukocytes that can phagocytose bacterial aggregates and the acidification of phagosomes upon lysosomal fusion.

The number of leukocytes at the site of infection was significantly lower in the Myd88-deficient larvae. This lower number of leukocytes at the site of infection at 4 dpi was associated with a significantly lower number of TUNEL-positive cells at this time point. This suggests that the lower number of leukocytes at the site of infection is due to reduced recruitment of these cells in *myd88*^-/-^ larvae rather than to an increased level of cell death. However, alternative explanations are possible, such as alterations in haematopoiesis that may be Myd88-dependent, or death of immune cells cell taking place outside the site of infection or at earlier stages. On the other hand, since our infection model only induces a very localized infection at the tailfin of larvae, only a relatively small number of leukocytes interact with the pathogens, which makes systemic effects unlikely. In addition, the total number of leukocytes has been shown to be similar in Myd88-deficient and wild-type larvae at 3 and 5 dpf [28, 36], so it seems most likely that the lower number of immune cell at the site of infection results from a reduced recruitment of leukocytes in the *myd88* mutant larvae.

In recent studies, a direct link has been demonstrated between reduced macrophage recruitment and increased susceptibility to *Mm* infection using zebrafish with genetic or pharmacologically induced macrophage deficiencies. The reduced migration of macrophages in these models results in an impaired supply of macrophages, so apoptotic macrophages in the granuloma are not engulfed by recruited cells. This causes secondary necrosis, breakdown of these granuloma and consequently spread of bacteria and accelerated extracellular growth [37–39].

However, at an early stage of infection (3 hpi), it has been shown that the recruitment of leukocytes towards an *Mm* infection site is not dependent on Myd88-mediated signalling [40]. Apparently, there is a difference in Myd88 dependency between initial and long-term leukocyte response to *Mm* infection. The long-term recruitment is likely to be dependent on pro-inflammatory mediators including cytokines and leukotrienes and tissue remodelling factors like matrix metalloproteinases, and the genes encoding these factors are strongly induced during the formation of granulomas in zebrafish larvae [28]. This induction has been shown to be dependent on Myd88 signalling [28], which may explain the lower number of leukocytes at the infected site in *myd88*^*-/-*^ larvae observed in the present study. Interestingly, the ratio between the number of dead cells and leukocytes present at the site of infection is higher in the mutant larvae, suggesting a higher percentage of cell death in recruited leukocytes. This may be a result of a higher bacterial load in the mutant larvae, since earlier observations have indicated that the lifespan of leukocytes is negatively correlated with the *Mm* load [24].

Using electron microscopy, we showed that the majority of bacteria in Myd88-deficient larvae were located extracellularly, most likely as a result of the reduced number of phagocytes present at the site of infection. The increased extracellular growth of *Mm* in *myd88*^-/-^ larvae found in this study is consistent with results obtained using comparable immune-compromised zebrafish models, using knockdown of the TNF and LTA4H expression [41]. In addition, the *myd88* mutant larvae showed more compacted aggregations of *Mm* at the site of infection than the wild types.

Performing ultrastructural analysis using TEM enabled us to quantitatively study the intracellular bacteria that resided in the immune cells of the mutant and the wild type. In previous studies, we showed that the largest fraction of intracellular *Mm* in wild-type larvae were observed as electron-dense aggregates [31], which are a result of efferocytosis [24]. Efferocytosis, which is defined as reuptake of cell debris and bacterial content by phagocytes upon death of an infected cell, is a crucial innate immune response in the defence against mycobacterial infection [42]. Interestingly, in the present study, we show that in mutant larvae, only a slight very small fraction was found as electron-dense aggregates and a larger fraction as aggregates without electron-dense content. We therefore conclude that the presence of electron-dense content, representing acidification of these compartments containing larger aggregates, resulting from fusion of the compartment with a lysosome, is highly Myd88-dependent. Restriction of bacterial burden is highly dependent upon acidification of *Mm* containing compartments [43], so the deficiency in this process most likely contributes to the increased bacterial growth in *myd88*^-/-^ larvae. Previously, we showed that DNA damage-regulated autophagy modulator 1 (Dram1) plays an important role in the lysosomal acidification of bacteria-containing compartments [27]. It was also shown in this study that the induction of dram1 expression upon *Mm* infection is Myd88-dependent. Therefore, we suggest that the reduced acidification of bacteria-containing compartments in the *myd88* mutant could be at least partly due to a compromised Myd88-Dram1 signalling pathway.

In summary, we have used a combination of light and electron microscopy applied to the tail fin *Mm* infection model in zebrafish larvae. We show that the inflammatory responses mediated by Myd88 affect the number of leukocytes present at the site of infection as well as the acidification of intracellular compartments. As a result, deficiency in Myd88-dependent signalling leads to an increased infection due to uncontrolled, mainly extracellular, mycobacterial growth.

## Data Availability

Data will be provided on request.
